# Comparative study of different liquid diets for dairy calves and the impact on performance and the bacterial community during diarrhea

**DOI:** 10.1038/s41598-022-17613-1

**Published:** 2022-08-04

**Authors:** Marina Gavanski Coelho, Gercino Ferreira Virgínio Júnior, Cristiane Regina Tomaluski, Ariany Faria de Toledo, Maria Eduarda Reis, Sophia Cattleya Dondé, Lucas William Mendes, Luiz Lehmann Coutinho, Carla Maris Machado Bittar

**Affiliations:** 1 Department of Animal Science, Luiz de Queiroz College of Agriculture, University of São Paulo, Av. Pádua Dias 11, Piracicaba, São Paulo 13418-900 Brazil; 2 Center for Nuclear Energy in Agriculture, University of Sao Paulo, Piracicaba, São Paulo 13400-970 Brazil

**Keywords:** Bacteria, Microbial communities, Gastrointestinal diseases, Nutrition, Microbiology techniques

## Abstract

The liquid diet composition can affect dairy calves' performance and diarrhea incidence. The effect of three liquid diets on performance, incidence of diarrhea, and microbial community during diarrhea occurrence in dairy calves were evaluated. At birth, 35 dairy calves (20 male and 15 female) were randomly assigned to one of three treatments—refrigerated whole milk (WM), acidified whole milk (AWM), and milk replacer (MR). Intake, fecal score, and rectal temperature were evaluated daily, and performance and blood parameters were evaluated weekly during the preweaning period. Fecal samples from diarrheic calves were collected, and one initial and one final sample for each episode were selected. The bacterial community was assessed by sequencing the V3-V4 region of the 16S rRNA gene on the Illumina MiSeq platform and analyzed using the DADA2 pipeline. Calves fed WM had higher body weight at weaning, average daily gain, body measurements, and concentration of blood metabolites. The AWM-fed calves had a lower rectal temperature and fever days. Moreover, the MR-fed calves had lower beta-hydroxybutyrate concentration and a higher incidence of diarrhea. The fecal bacterial community of diarrheic calves showed dissimilarity among the AWM and the other treatments. At the compositional level, we observed a higher abundance of *Fusobacterium* and *Ruminococcus* genera (AWM), *Prevotella* (WM), and *Lactobacillus* (MR). In the AWM and MR diarrheic calves' feces, we also observed some beneficial bacterial genera. The performance and incidence of diarrhea of dairy calves were influenced by the liquid diet consumed and the bacterial composition of diarrhea.

## Introduction

The preweaning period of calves usually ranges from 60 to 90 days, and most often, they are weaned at 90 days of age^1^. Thus, the liquid diet fed plays an important role since it provides the principal nutrients for the calf's development and substrate for microbial growth^2^.

The whole milk (WM) and milk replacer (MR) are the main liquid diets used in many countries, together with waste milk^3–5^. Whole milk is a high-quality option, but with considerable nutrient fluctuation, associated with higher cost. Waste milk also has a high production cost, which may be increased by the pasteurization equipament and process, whith the disadvantage of having an even high composition fluctuation compared to WM. On the other hand, MRs can be a more constant alternative, which can facilitate the management, but may present a challenge for young calves' intestinal health and performance according to its formulation^6,7^. In addition, the maturity of the gastrointestinal tract and intestinal digestion determine the digestibility and nutrient utilization by the calf. However, digestibility can be affected by both nutritional and microbiological quality^7^. Microbiological quality, in particular, is often neglected. Thus, newborn calves can be exposed daily to pathogens by feed intake, which may lead to infectious diseases, dangerous endotoxins, and gut disorders^8^.

Neonatal calf diarrhea is a gut disorder that causes many economic losses to dairy farmers because of mortality and morbidity, raising veterinary costs, and reducing performance. Thus, there is an imminent need to establish strategies to positively influence the gut microbiota composition and balance, which may reduce the occurrence and severity of diarrhea and improve calves' overall health and performance. The acidification process of the liquid diet is an interesting alternative to solve this problem. It prevents the growth of several pathogenic microorganisms, decreases digestive and diarrheal problems, improves performance and intestinal bacterial composition, and increases the population of beneficial microorganisms such as *Bifidobacterium* and *Lactobacillus*^1,8–11^.

The higher incidence of diarrhea in the first weeks of life can affect the homeostasis of gut microbial ecosystems^1^. The microbiota has been demonstrated to be a core piece for maintaining good health and the main functions of animal organisms, especially the gastrointestinal tract^2,12^. Slanzon et al.^13^ observed that calves with diarrhea and depressed had a higher relative abundance of *Streptococcus gallolyticus* and *Escherichia coli,* and calves with diarrhea, both active and depressed ones, had a lower relative abundance of *Bifidobacterium longum* compared to healthy calves. Gomez et al.^14^ also attributed the higher population of *Bifidobacterium* in the fecal microbiome to healthier calves.

Therefore, this study aimed to compare the performance, incidence of diarrhea, and metabolic parameters of Holstein dairy calves fed different liquid diets (WM, acidified WM, or MR). The second aim of this study was to compare the effect of the different liquid diets on the fecal bacterial community of these calves during the occurrence of diarrhea. We hypothesize that these differences are associated with the liquid diet composition; further, we believe that milk acidification may benefit calves' performance by gastrointestinal effects, as previously reported.

## Results

### Performance

**Figure 1:**
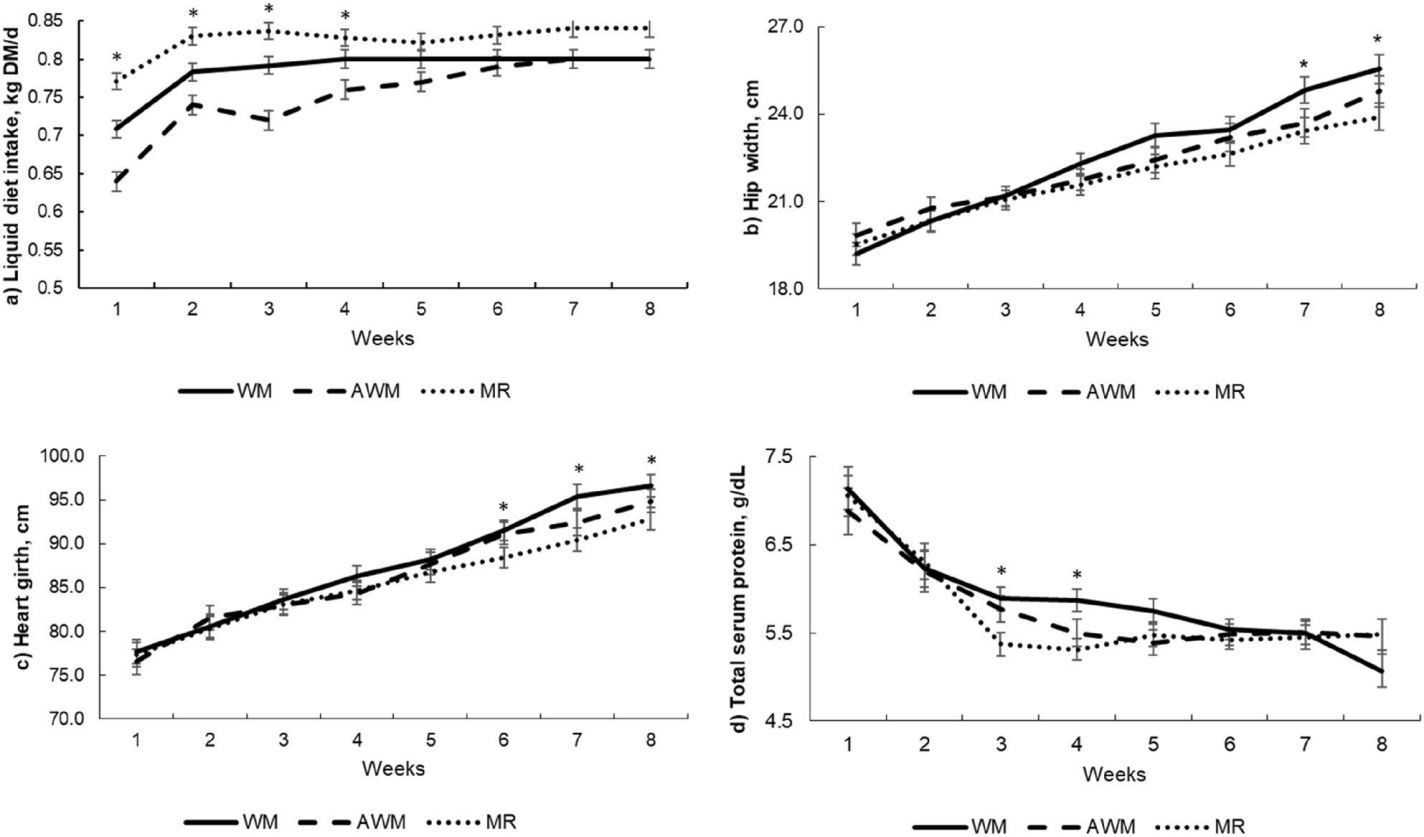
Intake, performance, and blood parameters of dairy calves fed different liquid diets. (**a**) Consumption of liquid diet; (**b**) hip-width; (**c**) heart-girth, and (**d**) total serum protein. *Denotes statistical difference P < 0.05 for the lowest values for AWM in (**a**); and WM higher than MR in (**b**)–(**d**).

The intake, ADG, and body measurement increased with age (*P* < 0.01; Table 1). Higher DM liquid diet intake was observed for MR-fed calves, followed by WM and AWM calves (Table 1; *P* < 0.0001); however, there was an age by liquid diet interaction effect, and differences occurred only until the 4th week of age (*P* < 0.004; Fig. 1a). Nevertheless, the liquid diet did not affect stater intake. Higher TDMI was observed for MR-fed calves than AWM (*P* < 0.04; Table 1). The feed efficiency also had an age effect with variations during the preweaning period (*P* < 0.01) and was the lowest for animals fed MR (*P* = 0.02). There was no difference in birth weight, but WM-fed calves had higher weaning weight (*P* < 0.01) and higher average withers height and ADG during the preweaning period (*P* < 0.01; Table 1). An age-liquid diet interaction effect was observed for hip-width and hearth-girth (*P* < 0.03 and *P* = 0.01, respectively), with WM-fed calves having higher hearth-girth and hip-width at the sixth and seventh week until weaning, respectively, than MR calves (Fig. 1b,c).

**Table 1:** Performance of preweaning dairy calves fed different liquid diets.

	Treatment			SEM	P-value		
	WM	AWM	MR		T	A	T × A
**Dry matter intake, kg/day**							
Liquid diet	0.785^b^	0.752^c^	0.825^a^	0.0055	< 0.0001	< 0.0001	0.004
Starter concentrate	0.21	0.17	0.19	0.04	0.66	< 0.01	0.85
Total	0.96^ab^	0.90^b^	0.99^a^	0.03	0.04	< 0.01	0.77
**Weight, kg**							
At birth	30.69	31.63	30.74	1.80	0.24	–	–
At weaning	70.54^a^	63.56^b^	61.15^b^	2.59	< 0.01	–	–
ADG, kg/day	0.70^a^	0.58^b^	0.54^b^	0.02	< 0.01	< 0.01	0.08
Feed efficiency	0.74^a^	0.70^a^	0.52^b^	0.06	0.02	< 0.01	0.32
**Body measurements, cm**							
Withers height	81.5^a^	79.1^b^	79.1^b^	1.08	0.01	< 0.01	0.54
Hip width	22.6^a^	22.2^a^	21.8^b^	0.35	0.05	< 0.01	0.03
Heart girth	87.6	86.4	85.5	1.22	0.08	< 0.01	0.01

*WM* whole milk, *AWM* acidified whole milk, *MR* milk replacer, *SEM* standard error of the mean, *T* treatment effect, *A* age effect, *T×A* treatment × age interaction effect (*P* < 0.05). ^a^, ^b^, ^c^ Means within a row with different superscripts are significantly different (P ≤ 0.05).

### Blood parameters

The selected blood parameters showed an age effect (*P* < 0.01; Table 2), with increasing BHB concentration and decreasing total serum protein (TSP), lactate, and glucose concentrations as calves aged. The lowest concentrations of lactate (*P* = 0.01) were observed for the AWM-fed calves (*P* < 0.01). The glucose concentration was the highest for the WM animals (*P* < 0.01). However, the lowest concentration of BHB was observed for MR-fed calves (*P* < 0.01). There was no difference among treatments for TSP, but an age-liquid diet interaction effect was observed, and at the third and fourth week of age, WM calves presented a higher concentration than MR-fed calves (Fig. 1d). (*P* = 0.01; Table 2).

**Table 2:** Blood metabolites of preweaning dairy calves fed different liquid diets.

	Treatment			SEM	P-value		
	WM	AWM	MR		T	A	TxA
Total protein, g/dL	5.88	5.78	5.74	0.105	0.55	< 0.01	0.01
Lactate, mg/dL	13.9^a^	11.2^b^	12.8^a^	0.47	0.01	< 0.01	0.51
Glucose, mg/dL	134.8^a^	111.0^b^	117.4^b^	3.28	< 0.01	< 0.01	0.27
BHB, mmol/L	0.129^a^	0.141^a^	0.081^b^	0.008	< 0.01	< 0.01	0.45

^1^*WM* whole milk, *AWM* acidified whole milk, *MR* milk replacer, *SEM* standard error of the mean, *T* treatment effect, *A* age effect, *T*×*A* treatment × age interaction effect (*P* < 0.05), *TSP* total serum protein. ^a^, ^b^ Means within a row with different superscripts are significantly different (P ≤ 0.05).

### Diarrhea and rectal temperature

**Figure 2:**
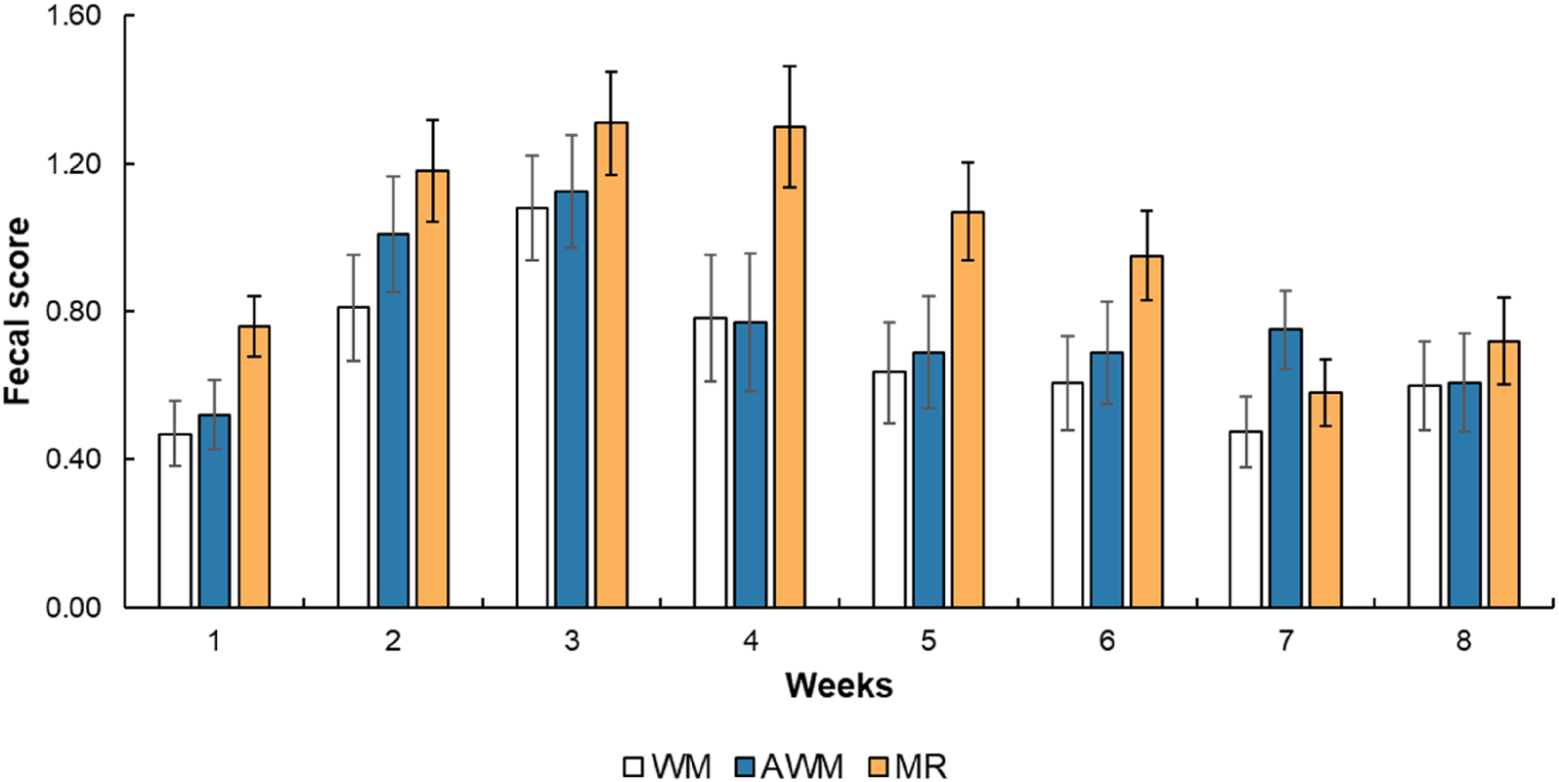
Fecal score of dairy calves fed different liquid diets. *Denotes statistical difference P < 0.05 for the lowest values for AWM and WM and higher than MR.

Age affected the fecal score, pH, and rectal temperature (*P* < 0.01). The fecal score increased until the third week of life but decreased after that (Fig. 2). In addition, MR-fed calves had higher fecal scores (*P* = 0.01; Table 3), days with diarrhea (*P* = 0.04), higher diarrhea rates at 0–56 days (*P* = 0.03) and 0–10 days (*P* = 0.04) as compared to WM animals. The WM calves had higher rectal temperatures than the AWM calves (*P* = 0.01). Also, the AWM calves tended to have a lower number of days with fever (*P* = 0.06).

**Table 3:** Diarrhea and health of preweaning dairy calves fed different liquid diets.

Indices	Treatment			SEM	P-value		
	WM	AWM	MR		T	A	T × A
Faecal score	0.68^b^	0.77^ab^	0.98^a^	0.08	0.01	< 0.01	0.43
Days with diarrhea	6.92^b^	9.40^ab^	12.69^a^	1.55	0.04	–	–
**Diarrhea rate, %**							
0–56 days	12.20^a^	16.80^ab^	22.80^b^	2.16	0.03	–	–
0–10 days	3.30^a^	5.00^ab^	16.90^b^	4.15	0.04	–	–
11–30 days	20.80	25.50	37.30	5.63	0.10	–	–
31–56 d	9.00	14.60	13.90	3.81	0.53		
Fecal pH	6.61	6.61	6.71	0.08	0.61	< 0.01	0.12
Haematocrit, %	27.91	28.09	25.99	0.83	0.14	0.96	0.21
Rectal temperature, °C	38.51^a^	38.35^b^	38.41^ab^	0.04	0.01	< 0.01	0.99
Days with fever	2.83^b^	1.04^a^	2.54^b^	0.55	0.06	–	–

*WM* whole milk, *AWM* acidified whole milk, *MR* milk replacer, *SEM* standard error of the mean, *T* treatment effect, *A* age effect, *T*×*A* treatment × age interaction effect (*P* < 0.05). ^a^, ^b^ Means within a row with different superscripts are significantly different (P ≤ 0.05).

### Fecal bacterial community

**Figure 3:**
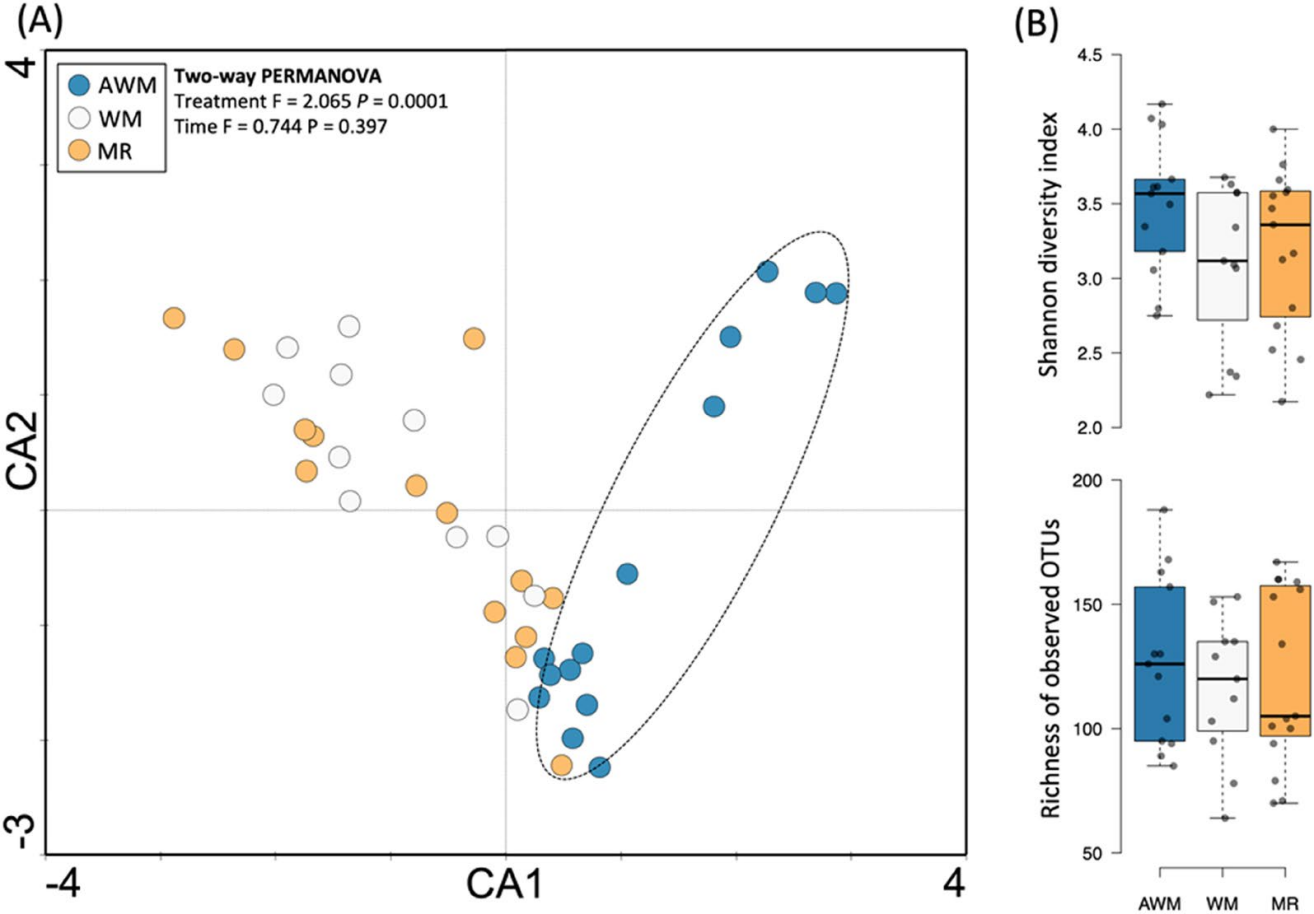
Structure and microbial diversity in diarrheal fecal samples from dairy calves fed different liquid diets. (**A**) Correspondence analysis (CA); (**B**) diversity indexes, Shannon and Richness.

The bacterial community structure of fecal diarrhea samples showed dissimilarity among samples from the AWM group and the WM and MR groups (Fig. 3a; *P* < 0.01). The correspondence analysis (CA) only showed differences between indices not influenced by the different liquids (Shannon, *P* = 0.632; and Richness*, P* = 0.720; Fig. 3b). Also, no differences were observed between the “initial and final” time points or “episodes” of diarrhea (*P* > 0.05). Given this result, the following data will be presented as a mixed result.

**Figure 4:**
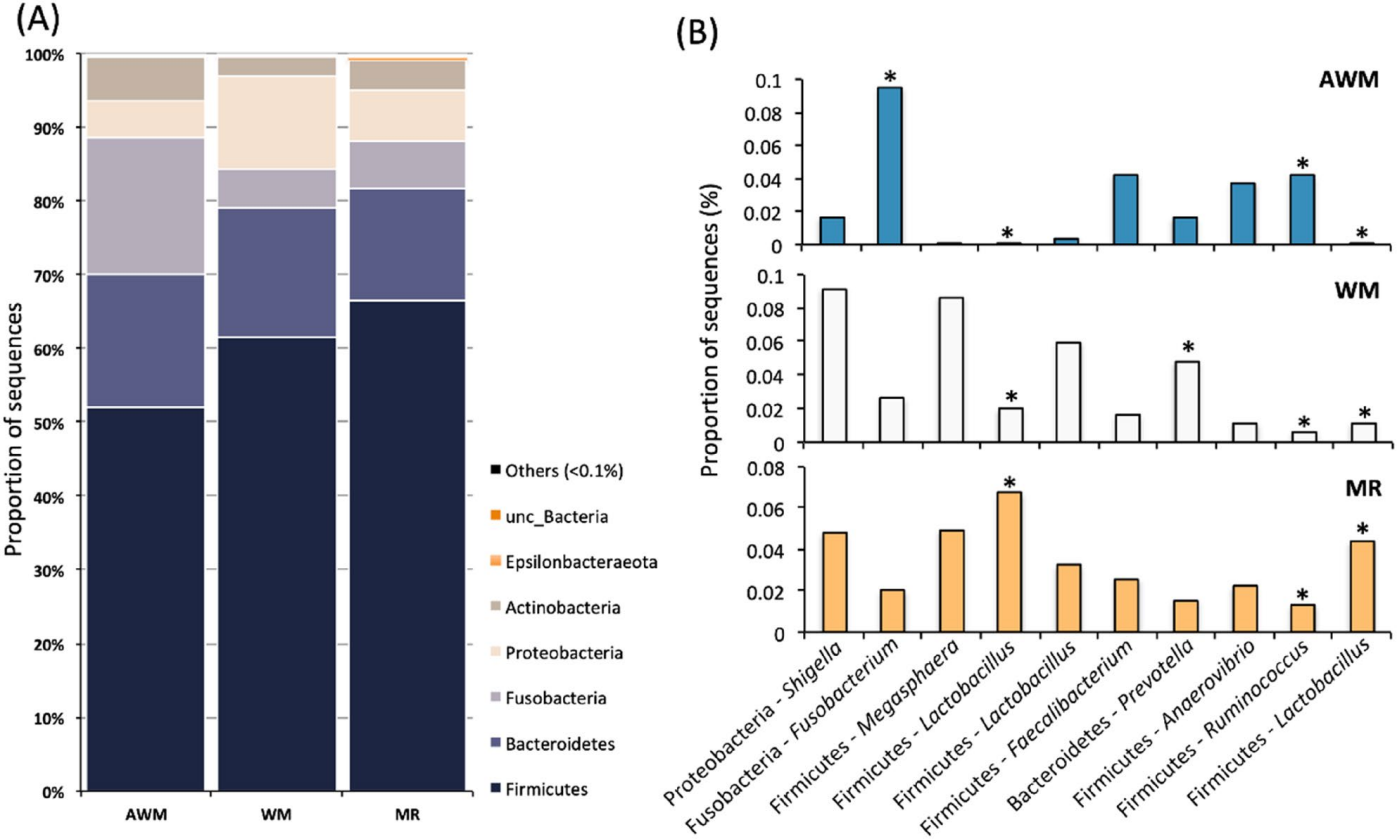
Bacterial composition in diarrheal fecal samples from dairy calves fed different liquid diets. (**A**) General phylum abundance; and (**B**) Top 10 most abundant ASVs. *Denotes statistical difference of P < 0.05 for higher abundance of *Fusobacteirum* for AWM; higher abundance in MR and the lowest in AWM for LL*actobacillus*; higher abundance of *Prevotella* for WM; and highest abundance of *Ruminococcus* in AWM and lowest in MR.

The general phylum abundance is represented in Fig. 4a. Overall, over 60% of the sequences were classified as Firmicutes, followed by Bacteroidetes (17%), Fusobacteria (10%), Proteobacteria (8.1%), Actinobacteria (4.2%), and Epsilonbacteraeota (0.2%). The ten most abundant ASVs (Amplicon Sequence Variant) were represented in bar graph in Fig. 4b. The abundance of *Fusobacterium* was higher in AWM compared to the other treatments (*P* = 0.049). Three ASVs were classified as *Lactobacillus*, but only two were significantly different among the treatments, the most abundant in MR and the lowest in AWM (*P* < 0.001 and *P* = 0.015, respectively). The ASV classified as *Prevotella* had a higher abundance in WM-fed animals than in the other treatments (*P* = 0.031). Furthermore, the ASV classified as *Ruminococcus* was most abundant in AWM and less abundant in MR (*P* = 0.037).

**Figure 5:**
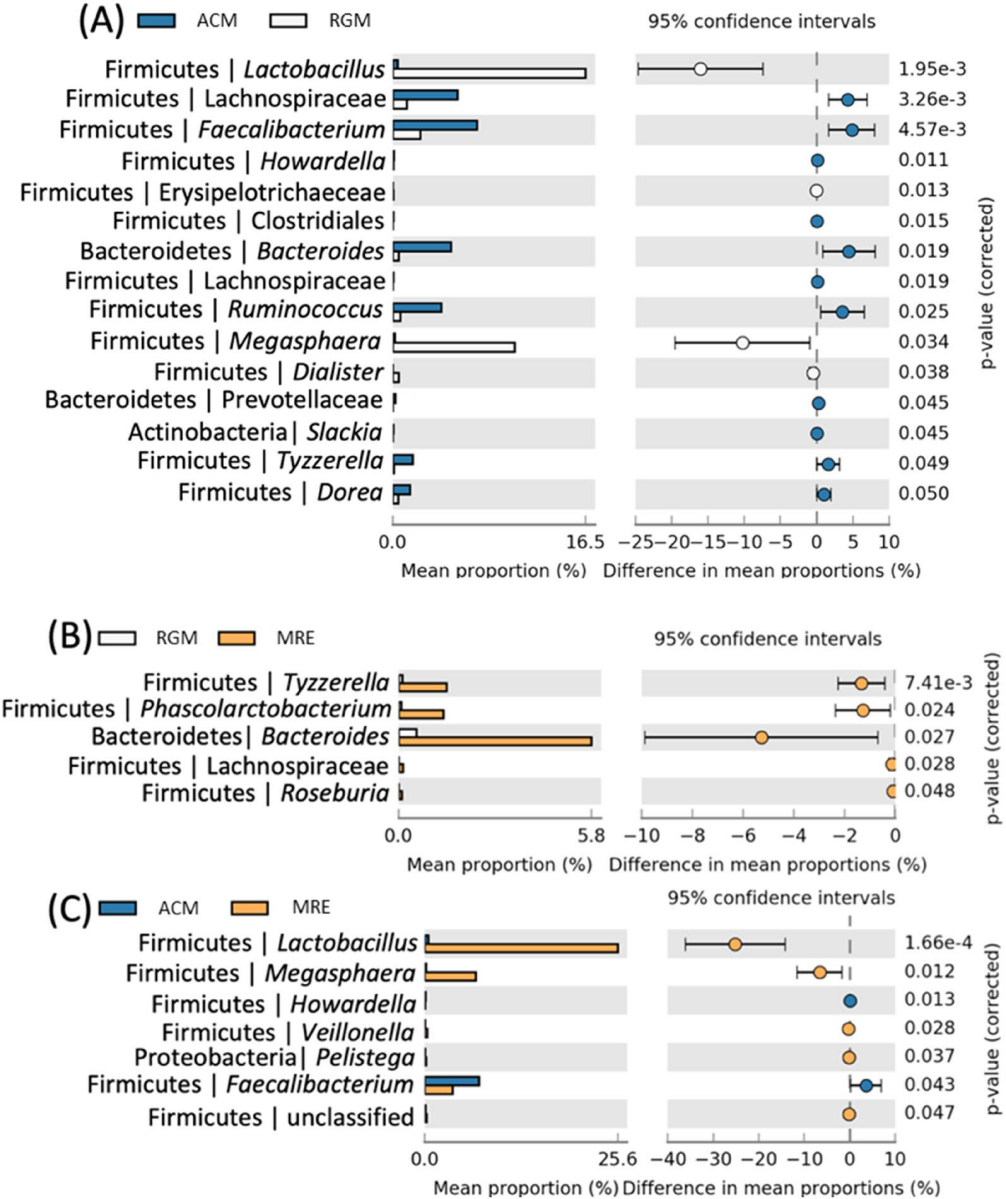
Bacterial genera composition in diarrheal fecal samples from dairy calves fed different liquid diets. Scatter-plot based on Welch's *t* test with Benjamini–Hochberg correction constructed in STAMP (P < 0.05). (**A**) differences between AWM and WM; (**B**) differences between WM and MR; and (**C**) AWM and MR.

In a pairwise comparison at the genus level on the bacterial composition, there were significant differences between AWM and WM-fed calves for 15 different ASVs (Fig. 5a), but only those classified as *Lactobacillus*, *Erysipelotrichaeceae*, *Megasphaera,* and *Diallister* were higher in WM-fed animals. In the WM versus MR (Fig. 5b) comparison, all five ASVs were significantly higher in MR-fed animals. Finally, when comparing AWM and MR (Fig. 5c), out of the seven statistically different ASVs, only *Howardella* and *Faecalibacterium* were higher in AWM-fed animals.

## Discussion

### Performance

Whole milk should be the first feeding option for dairy calves in the preweaning period^2^. The natural formulation of WM is complete, including several hormones, antibodies, glycans, glycoconjugates, antimicrobials, and an excellent substrate source for gut microbiota growth^15^. Our study shows that this complete and complex composition associated with adequate feeding volume (6 L/day) can promote a superior performance compared to MR. Whole milk has advantages over MR because of the higher energy content and a better balance of nutrients, mainly aminoacids^7,16^. Besides that, adequate nutrition might stimulate an immune response in calves during the challenging period of diarrhea occurrence and lead to a decrease in the rate of disease^17^. The WM-fed calves had a more significant average daily gain, higher withers height, greater body weight at weaning, and greater hip-width and heart-girth in the last weeks of the preweaning period. This means that the calves fed WM gained more weight, were more efficient and presented improvements in all the evaluated body measurements. According to Coelho et al.^10^, milk acidification is a method of preserving the microbiological quality of milk, as lower pH is unfavorable to the survival and growth of pathogenic microorganisms. Todd et al.^9^ suggested that acidification changes milk palatability enough to decrease milk consumption without affecting performance. In our study, acidified milk palatability may have influenced intake in the first 3 weeks of life, but after that, intake was the same as observed for WM-fed calves. However, although efficiency was similar to that of calves fed WM, calves on the AWM diet presented ADG and final weight similar to that of calves fed MR. The combination of the type of acid used, pH, and volume of the liquid diet most likely influences the liquid diet intake in the first weeks of life. The acidification process with formic acid at pH between 4 and 4.5 has also been shown to limit the voluntary intake of calves consuming MR ad libitum by approximately 1 L/day^18^. However, no differences in intake were observed in the preweaning period between WM and WM acidified at pH 4.2, at a 6 L/day volume using lactic acid^10^. In studies of restricted feeding (8% of BW) of an acidified diet (pH 4.8) with formic acid, no decrease in intake was observed compared to control^19,20^. Furthermore, the acid type should be carefully considered, as Zou et al.^11^ observed higher inflammation scores in the jejunum and ileum in calves fed the liquid diet acidified with formic acid. No such information was found for the other acids used.

Literature suggests that the MRs used in intensive feeding should be higher than 24% protein^21^. However, most formulations do not have this protein level and include ingredients of non-milk source^7^, which reduces the digestibility and utilization of the liquid diet in the first weeks of age since the gastrointestinal tract is not fully developed to digest this type of nutrient^7,22^. The MR used in this study contains non-milk fat sources, as mentioned by Virginio Junior et al.^1^. The type of liquid diet can affect the positive aspects of biologically active factors in WM that generate lactocrine effects, which may even attribute greater long-term production compared to MR^23^. Recent studies suggest ingesting nutrients from WM or MR alters phenotypic expression for long-term milk production^24^. In addition, Górka et al.^25^ observed that calves fed with MR had less intestinal development, organ weight, and microvilli length than calves fed WM. The authors also concluded that changes in intestine development, in addition to influencing performance data, interfere with the general metabolite state, which may indirectly affect the proper functioning of the immune system. This corroborates negatively with data on blood metabolites and diarrhea for MR treatment.

### Blood metabolites

In a meta-analysis of digestibility data of different liquid diets based on WM or MR, Quigley et al.^22^ observed that the digestibility of some nutrients (i.e., DM, N, and fat) reached its maximum at 30 days of age. Eventually, any gut disorder (i.e., diarrhea) or liquid diet quality affecting the intake or digestibility of the diet could negatively affect the performance of animals. This information could explain why animals in the AWM and MR groups showed lower performance and plasma glucose or even why the MR group had lower serum protein in weeks 3 and 4. According to Quigley et al.^26^, BHB is an indirect indicator of ruminal development, and its concentration increases according to the concentrate intake. However, there were no differences in starter intake, suggesting that the liquid diet composition explains differences in performance and selected metabolites concentrations. Higher glucose for calves fed WM may be explained by the higher lactose reaching the intestine for absorption by the sampling time because of the clotting process and digestibility of this liquid diet; while the higher BHB for calves fed the higher fat content of both could explain WM and AWM as compared to MR.

On the other hand, the liquid diet can also affect the plasma concentration of insulin and IGF-1, which are important in stimulating the proliferation of rumen epithelial cells^27,28^. The study by Górka et al.^25^ reported a shorter papilla length in the cranial dorsal sac in MR-fed calves than in calves fed WM and noted positive relationships between reticulo-rumen weight and small intestine weight or with enzymatic brush border activity. These effects would also explain the low BHB concentration for the MR group since there was no difference in starter intake among treatments. This indicates that the lower performance associated with higher enteric disorders (i.e., diarrhea) and lower concentration of metabolites negatively affected the ruminal development of calves in this group.

### Diarrhea and rectal temperature

The effect of the different liquid diets on diarrhea was well pronounced. Although animals in the WM group had a higher rectal temperature and a higher number of days with fever, the rate and days with diarrhea were lower. According to Todd et al.^20^, acidification of a liquid diet tends to increase feces fluidity but is not associated with other gut infection signs. This information can relate to our study since animals had intermediate values for diarrhea findings, but rectal temperature and days with fever were lower. Güler et al.^22^ demonstrated that although fecal consistency is more fluid in calves consuming an acidified diet, the number of veterinary interventions is lower, suggesting that acidification of the digestive tract may promote the growth of beneficial microorganisms and inhibit the proliferation of pathobionts^1^.

Furthermore, the effect of acidification on the intestinal tract may explain why we have observed dissimilarity in the bacterial community structure of feces from diarrheic calves compared to the WM or MR group. All treatments increased fecal scores in the 2nd and 3rd weeks of life; however, MR-fed calves had higher fecal scores than WM, without differing from AWM. Nevertheless, it is important to note that the diarrhea rate was higher for calves fed MR than WM and intermediate for AWM-fed calves for the entire period and the first 10 days of age. This higher and intermediate diarrhea rate negatively impacted the performance of calves fed MR and AWM, respectively. According to Cho and Yoon^29^, the first 21 days of life are challenging because calves are highly susceptible to enteric infections, one of the main causes of mortality preweaning. The enteric infections are associated with dysbiosis in the gut microbiome, inflammation of gut tissue^12,30^, and lower animal performance, as observed in MR treatment.

### Fecal microbiome during episodes of diarrhea

The fecal bacterial community in diarrheic calves showed a higher abundance of ASVs associated with the genus *Prevotella*, which strains have been mentioned in some reviews as pathobionts and associated with dysbiosis intestine inflammation in humans^31,32^. Despite this association, in ruminants, the genus *Prevotella* plays an important role in the degradation of several nutrients^33^, and its increase in feces and rumen of calves is associated with the consumption of concentrates, as observed in previous studies from our lab^1,34,35^. However, animals fed WM have been associated with a beneficial gut microbiota consisting of *Lactobacillus* and *Faecalibaterium*^1^. The study by Virginio Junior et al.^2^ used 15 healthy animals from our study (5 from each treatment), and although the diarrhea samples in our study did not show a high abundance of these microbial groups, their effects during the preweaning period may have helped to fight other pathogenic bacteria, in addition to bringing health benefits to the animals. The higher abundance of ASVs associated with the genera *Fusobacterium*, *Faecalibacterium*, *Ruminococcus*, and *Bacteroides* may indicate that there is indeed a benefit from acidification for the hindgut tract during diarrhea episodes since these bacteria are butyrate-producers. Butyrate is a source of colonocytes growth and indicates a healthy intestinal environment^36–39^. A recent study attempted to improve calf health by manipulating early gut microbial composition with oral supplementation of *Faecalibacterium prausnitzii*^40^, a bacterium negatively associated with calf diarrhea^38^. Oral administration of *F. prausnitzii* during the first week of life effectively reduced the incidence of diarrhea and calf death related to diarrhea in preweaned calves during the first 7 weeks of life^40^.

A higher incidence of diarrhea for the MR animals appears to be associated with the MR composition. Virginio Junior et al.^1^ mentioned that the composition of MR may have induced a greater abundance of pathobiont bacteria in the calves' hindgut. One interesting point is the higher abundance of ASVs related to *Lactobacillus* during diarrhea. The abundance of this genus may minimize and control the effects of diarrhea and other infections since these animals also had a higher number of days with fever. In addition, *Lactobacillus* strains were associated with a lower incidence of enteric infections, disorders, and greater stimulation of the mucosal immune system^41,42^.

This was also a finding in a study by Slazon et al.^13^, as calves with diarrhea had a higher population of *Lactobacillus*. These authors developed a model to predict gastrointestinal disease based on fecal microbiome composition, and the presence of *Eggerthella lenta*, *Bifidobacterium longum*, and *Collinsella aerofaciens* were associated with a healthy clinical outcome. The presence of *E. coli* and *Lactobacillus* species had the highest coefficients positively associated with GI disease prediction.

The abundance of *Tyzzerella* has not yet been related to any health or disease status in dairy calves, but in humans, the abundance of this genus has been associated with Crohn's disease patients or poor-quality diets^43,44^, which may indicate a possible negative occurrence in the intestines of dairy calves. The same occurs for the *Veillonella* genus, associated with producing toxic compounds (i.e., NH_3_, H_2_S, amines, and others) by the protein degradation^45^.

In conclusion, the performance and health of dairy calves were influenced by the liquid diet intake and the bacterial composition of diarrhea. The low abundance of bacteria pathobionts in WM may indicate that other pathogenic microorganisms cause the diarrheal episodes, and perhaps the milk's bioactive components prevent opportunistic bacteria, as opposed to what was observed in MR. Furthermore, acidification may induce beneficial and mucosal protective bacteria abundantly in diarrheal episodes.

Whole milk was the best choice of liquid diet, promoting higher performance and less negative impact on animal health based on microbiome analysis. Acidified milk may be an interesting alternative for situations with difficulty refrigerating WM, with the intermediary occurrence of diarrhea but lower performance, even though microbiota during diarrhea episodes were beneficial. In addition, the use of MR for preweaning calves should be used carefully, especially during the first 3 weeks of life, since the formula may have carbohydrates and protein of vegetable origin and low digestibility.

## Materials and methods

All procedures with calves were performed in accordance with the relevant guidelines and regulations approved by 'Luiz de Queiroz' College of Agriculture, University of São Paulo, Brazil. All the experimental procedures were approved following the ethics by the Institutional Animal Care and Use Committee in the "Luiz de Queiroz" College of Agriculture, University of São Paulo, Brazil (Protocol no. 2018.5.586.11.7). We confirm that this study was carried out in compliance with the Animal Research: Reporting of In Vivo Experiments (ARRIVE) guidelines.

This study was conducted at Experimental Calf Facility of the ‘Luiz de Queiroz’ College of Agriculture, University of São Paulo, Brazil, from February to May 2019. During this period, the average temperature was 23.2 ± 1.7 °C, max 29.6 ± 1.3 °C, and min 17.1 ± 2.3 °C, and the average relative humidity was 78.5%.

### Animal, experimental design, and treatments

Thirty-five newborn Holstein calves (20 males: 32.84 ± 2.4 kg; and 15 females: 28.22 ± 1.6 kg; mean ± SD) were used in a randomized complete block design. Calves were blocked according to sex, birth date, and birth weight. Calves were sourced from the university dairy farm and a nearby commercial farm and were transported to the experimental calf facility immediately after birth.

All calves were fed 10% of birth weight of high-quality colostrum (> 50 g IgG/L) within the first 6 h of life^46^. The passive transfer was evaluated at 48 h after colostrum feeding. The mean serum protein (TSP) was 6.0 g/dL, suggesting an adequate passive immune transfer for all the calves, with a cut-off point of 5.5 g/dL considered^46^.

Calves were fed 6 L/day of diet liquid supplied in open buckets divided into 2 meals (7h00 and 17h00), according to treatments: (1) refrigerated whole milk (WM; n = 12); (2) acidified whole milk (AWM; pH 4.5; n = 10); or (3) commercial MR (MR; n = 13). Whole milk was collected every 2 days in the milking parlor, half was refrigerated at 5 °C, and the other half was acidified until pH 4.5. The acidification process was done according to Virgínio Júnior et al.^1^. The milk was acidified at least 12 h adding formic acid (Formic acid 85%, Dinâmica Química Contemporânea Ltda, SP, Brazil) before feeding and kept at room temperature.

The MR (Sprayfo Azul, Sloten from Brazil Ltda, SP, Brazil; Table 4) was diluted to 14% solids in potable water. Average milk composition was: 3.8% fat, 3.3% protein, 4.4% lactose, 12.6% total solids, and somatic cell count (SCC) of 340,000 cells/mL. All liquid diets were heated to 38–40 °C before feeding and supplied from 2 days of life until weaning (56 days). All calves were gradually weaned at 57 days of age, reducing the liquid diet supply by 1 L per day until complete weaning at 62 days.

**Table 4:** Chemical composition of the calf starter and milk replacer.

	Calf starter^1^	Milk replacer^2^
Dry matter, g/kg	900.9	964.8
Ash, g/kg DM	73.0	87.2
Crude protein, g/kg DM	232.2	229.0
Crude fat, g/kg DM	29.6	187.8
NDF, g/kg DM	168.6	11.3
NFC, g/kg DM	510.2	510.4

^1^Ag Milk Agroceres Multimix Nutricao Animal Ltda., Rio Claro, SP, Brazil; ^2^Sprayfo Azul, Sloten from Brazil Ltda, SP, Brazil: 25,000 UI Vit A, 5000 UI Vit D3, 336 UI/kg Vit E, 300 μg/kg Se, 10 mg/kg Cu and 90 mg/kg of Fe; Ingredients composition: Whey, lactose-free whey, concentrated whey protein, lipids of vegetable origin, hydrolyzed wheat gluten, folic acid, calcium pantothenate, vitamins (A, D3, E, K, C, B_1_, B_2_, B_6_, B_12_), biotin, niacin, iron sulfate monohydrate, copper sulfate pentahydrate, magnesium sulfate, manganese sulfate monohydrate, sodium selenite, potassium iodide, zinc sulfate monohydrate, probiotic additive; *NDF* Neutral detergent fiber, *NFC* Nonfiber carbohydrate.

### Calves housing and intake

Calves were housed in individual suspended cages (113 × 140 cm) with sawdust beds in a ventilated barn during the first 14 days. On day 15, calves were relocated to an outdoor individual wood shelter (1.35 m height, 1 m width, and 1.45 m depth) and contained by a chain belt attached to a thin chain (2 m long), allowing an adequate walking area, but with no physical contact with other calves. Calves had free access to water and a commercial calf starter (Racao Bezerra Ag Milk Agroceres Multimix Nutricao Animal Ltda., Rio Claro, SP, Brazil; Table 4). The calf starter was supplied before the morning liquid diet feeding and was available until the next day when the orts were weighed to measure daily intake.

### Sampling and laboratory analysis

The calf starter and MR samples were collected monthly to determine chemical composition. Feed samples were dried (MA035, Marconi, Piracicaba, São Paulo, Brazil) at 55 °C for 72 h and ground through a 1-mm screen Wiley mill (Marconi). The DM was determined by drying the samples in an oven at 105 °C for 24 h (AOAC method 925.40) and ash by incinerating the samples in a muffle furnace at 550 °C for 4 h (AOAC method 942.05). The detergent fiber analysis was used to determine NDF^47^ concentrations in an Ankom 2000 fiber analyzer (Ankom Tech. Corp., Fairport, NY). Heat-stable α-amylase and sodium sulfite were included in the NDF analysis. Total nitrogen concentration was determined using the Leco TruMac N apparatus (Leco Corporation, St. Joseph, MI; AOAC method 968.06). The crude protein was calculated by multiplying the total nitrogen by 6.25. The ether extract concentration was determined using petroleum ether (AOAC method 920.39). The NFC of the diets were estimated according to the following equation: NFC (%) = 100% − (% NDF + % CP + % fat + % ash), according to Mertens (1997). Bulk milk samples were collected biweekly during the overall experiment period for TS analyzed by Fourier-transform infrared spectroscopy^48^ and for SCC by flow cytometry (Clínica do Leite, Piracicaba, Brazil). The WM composition was 3.84% fat, 3.29% protein, 4.44% lactose; 12.57% total solids %, and 326 × 10^3^/mL of somatic cell count (SCC). The calf starter and MR chemical composition are described in Table 4.

### Body measurements and blood parameters

Calves were weighed at birth and weekly until the eighth week of age using a mechanical scale (ICS-300, Coimma Ltda., Dracena, SP, Brazil), always before the morning feeding. Body measurements were also recorded weekly. Wither height and hip-width were measured with the aid of a ruler with a scale in centimeters (Carci, São Paulo, SP, Brazil), and heart girth, a measuring tape was used with a scale also in centimeters (Bovitec, São Paulo, SP, Brazil).

Blood samples were obtained weekly by venipuncture of the jugular vein, always 2 h after the morning feeding. Samples were collected in three tubes (Vacuette do Brasil, Campinas, SP, Brazil) containing sodium fluoride as an antiglycolytic, potassium EDTA as an anticoagulant, and K_3_ EDTA, both to obtain plasma and a clot activator to obtain serum. Tubes were immediately placed on ice and transported to the laboratory.

An aliquot of blood from the K_3_ EDTA tube was used for capillary hematocrit using a microhematocrit centrifuge at 2000×*g* for 10 min (model SPIN 1000, Microspin, Sao Paulo, Brazil). Samples were centrifuged at 2000×*g* for 15 min at 4 °C to separate the serum and plasma and then stored at − 10 °C for subsequent analysis. The metabolic indicators were determined in the Automatic System for Biochemistry (SBA-200, CELM, Barueri, SP, Brazil). Specific commercial enzymatic kits (Labtest Diagnóstica S.A., Lagoa Santa, MG, Brazil) were used to analyze plasma glucose (ref. 85), total serum protein (ref. 99), and lactate (ref. 116). Beta-hydroxybutyric acid (BHB) was determined using the RANBUT enzyme kit (Ref.: RB1007; RANDOX Laboratories—Life Sciences Ltd., Crumlin, UK).

### Fecal score and pH

The fecal score was monitored daily regarding the fluidity of feces: (0) regular and firm; (1) soft; (2) aqueous; (3) fluid, according to Calf Heath Scoring Criteria previously published by The University of Wisconsin (Madison) by McGuirk^49^. The diarrhea episode was considered when the calves presented fecal score ≥ 2 for more than 1 day^50^. The diarrhea rate was calculated according to the formula adapted from Li et al.^51^: Diarrhea rate = (days with diarrhea ÷ days evaluated) × 100.

Calves with a score ≥ 2 received an oral rehydration solution (1 g of potassium chloride, 80 g of dextrose, 4 g of sodium bicarbonate, and 5 g of sodium chloride per liter of warm water) at 11 h. Calves’ rectal temperature was measured daily using a digital thermometer, and fever was considered when calves presented more than 39.4 °C. Health problems were monitored and treated according to veterinary recommendations. Fecal pH was measured weekly, 2 h after the morning feeding, using 4 g of feces added to 4 mL of deionized water, using a digital pH meter (tec-5 model, Tecnal Ltda.), according to Channon et al.^52^.

### Fecal bacterial community evaluation of animals affected by diarrhea

#### Fecal diarrhea sampling

For two consecutive days, fecal samples were collected from calves with a fecal score ≥ 2 (positive case for diarrhea). Thus, we started sampling on the second day of diarrhea and continued daily until the feces presented a score ≤ 1. Then, the episode was delimited by at least 2 days of fecal score zero. On average, calves began to have diarrhea on day 14.5.

Samples were collected manually with gloves directly from the rectal ampoule, and gloves were discarded at each collection to avoid cross-contamination between samples. If no material was collected at the time of collection, that day of collection was not considered but continued the next day if the score remained ≥ 2. About 2 g of feces were collected, placed in sterile tubes, and immediately frozen at − 20 °C.

A total of 117 samples were collected from 27 calves throughout the experimental period. To analyze the fecal bacterial community of diarrheal animals, we randomly selected 4 calves from these samples per treatment. We selected animals with at least two episodes of diarrhea as a selection criterion. An exception was made for the AWM group, as only three animals met the selection criteria. The fourth animal for this treatment was selected because it had a longer period of diarrhea (6 days).

After selecting the animals, we selected specific samples from each diarrheal episode. For animals with a longer duration of diarrhea (> 3 days), we selected one sample from the initial day of episode and one sample from the final day collected. Only samples from the initial day of the episode were selected for calves that had diarrhea for < 3 days. In total, we selected 42 samples to analyze the fecal diarrheal bacterial community. On average age, diarrhea samples were collected on days 13.16 and 22.17, respectively, for the beginning and the end of the first episode, and on days 24.36 and 34, respectively, for the beginning and the end of the second episode.

#### DNA extraction, library preparation, and sequencing

DNA extraction from fecal samples was performed with the QIAamp^®^ Fast DNA Stool Minikit extraction (Qiagen, Hilden, Germany), following the modifications suggested by Yu and Morrison^53^.

The libraries were prepared following Illumina's recommendations and described in the Virginio Junior et al.^1^. The primers used for locus-specific amplification of bacteria flank the V3–V4 region. The overhang sequence of adapters is included in locus-specific primers. The full-length primers sequence were: 16S Amplicon PCR Forward Primer: 5′ TCGTCGGCAGCGTCAGATGTGTATAAGAGACAGCCTACGGGNGGCWGCAG; 16S Amplicon PCR Reverse Primer: 5′ GTCTCGTGGGCTCGGAGATGTGTATAAGAGACAGGACTACHVGGGTATCTAATCC.

Sequencing was performed in the Illumina Miseq system (Illumina, San Diego, CA, USA) and produced readings were 2 × 250 bp.

#### 16S rRNA sequences processing

The 16S rRNA gene sequencing data were processed using QIIME2 version 2019.10. Firstly, the paired-end sequences were merged using PEAR^54^, the sequences were demultiplexed, and quality control was carried out using DADA2^55^, using the consensus method to remove any remaining chimeric and low-quality sequences. After filtering, approximately 2.47 million high-quality reads (on average, ~ 60,000 reads per sample). Afterward, the samples were rarefied to 21,700 sequences, following the number of the lowest sample, and singletons and doubletons were removed. The taxonomic affiliation was performed at 97% similarity using the Silva database v. 132^56^, and the generated matrix was further used for statistical analyses.

### Statistical analysis

#### Performance, health, and metabolism data analysis

The experimental design was a randomized block, and calves were allocated into blocks according to birth weight, date of birth, and sex. Differences in weight within blocks were no more than 2 kg, while the age of the calves varied by a maximum of 15 days. The growth and intake data, blood metabolites, and fecal score were analyzed as time-repeated measures using the MIXED procedure of the SAS statistical package (version 5.0, SAS Institute Inc., Cary, NC, USA) for mixed models. The model included treatment, week (age of calves), and the interaction between treatment and week as fixed effects. The block effect was included in the model as a random effect and week as a repeated measure (model 1), and the subject of the repeated measures used was animal (treatment). Statistical model 1: Yijk = μ + Ti + bj + eij + Sk + (TS)ik + eijk, where: Yijk—response variable, μ—general average, Ti—fixed effect of treatment, bj—random block effect, eij—residual error (A), Sk—fixed age effect of calves (week of data collection/sample), (TS)ik—fixed effect of the treatment × age interaction, eijk—residual error (B). The covariance structures “compound symmetry, heterogeneous compound symmetry, autoregressive, heterogeneous autoregressive, unstructured, banded, variance components, toeplitz, antidependence and heterogeneous Toeplitz” were tested and defined according to the lowest value obtained for “Akaike's Information Criterion corrected” (AICC).

For data pooled to create a single measure, such as diarrhea days and days with fever, PROC MIXED of the SAS statistical package was used according to model 2. The model included treatment as a fixed effect and block as a random effect, and calf within treatment was included as a random effect. The means were obtained for all the response variables through the LSMEANS command. The Tukey's test performed the comparisons among the treatments when there was significance in the analysis of variance. Statistical model 2: Yijk = μ + Ti + bj + eij, where: Yijk—response variable, μ—general mean, Ti—fixed effect of treatment, bj—random block effect, eij—residual error]. For all the analyses, significance was declared at *P* < 0.05.

#### Fecal bacterial community data analysis

The statistical analyses were performed by comparing the treatments and the time. Data were presented together when the effect of the time was negligible. Initially, the data were checked for normal distribution using the Shapiro–Wilk test and homogeneity of variance using Levene's test, which indicated non-normal distribution. Thus, correspondence analysis (CA) assessed the bacterial community structure using Canoco 5 (Biometrics, Wageningen, The Netherlands). We used two-way PERMANOVA^57^ to test if treatments and time harbored significantly different microbial communities. Shannon's diversity and richness measurements were calculated based on the taxonomic matrix at the OTU level using PAST 4.01 software^58^. To compare the composition of the bacterial communities among treatments, we used the Statistical Analysis of Metagenomic Profile (STAMP) software^59^. The OTU table at the phylum and genus level generated from QIIME 2 was input. P-values were calculated using a two-sided Tukey–Kramer test, and the correction was made using the Benjamini–Hochberg false discovery rate^60^.

## Data Availability

All raw DNA sequence reads were deposited in NCBI's Sequence Read Archive under BioProject PRJNA734655. The other data presented in this study are available on request from the corresponding author. The data are not publicly available due to restrictions by the research group.
